# Expert Knowledge-Infused Learning for Indoor Radio Propagation Environment Digital Twins

**DOI:** 10.3390/s26072199

**Published:** 2026-04-02

**Authors:** Haotian Wang, Lili Xu, Yu Zhang, Tao Peng, Wenbo Wang

**Affiliations:** 1 Key Laboratory of Universal Wireless Communications, Ministry of Education, Beijing University of Posts and Telecommunications, Beijing 100876, China; htwang@bupt.edu.cn (H.W.); wbwang@bupt.edu.cn (W.W.); 2 Research Institute of China United Network Communications Co., Ltd., Beijing 100176, China; xull82@chinaunicom.cn (L.X.); zhangyu9@chinaunicom.cn (Y.Z.)

**Keywords:** deep learning, digital twin, indoor radio communication, path loss prediction, radio propagation

## Abstract

Digital Twin (DT) technology, which enables the simulation, evaluation, and optimization of physical entities through synchronized digital replicas, has attracted increasing attention in the context of wireless networks. Among the various components involved, the radio propagation environment is fundamental to communication performance, making its accurate digital replication a critical challenge. This paper focuses on constructing a high-precision radio propagation environment DT using deep learning (DL) methods. While data-driven DL has become a mainstream solution for signal propagation prediction in DTs, its performance depends heavily on the model’s ability to learn intrinsic propagation patterns from data. Owing to the complex interactions between wireless signals and environmental obstacles, conventional DL models often struggle to efficiently capture implicit propagation laws solely from raw data. To address this issue, we propose a general methodology for incorporating expert knowledge of radio propagation into DL frameworks. Building upon the widely adopted encoder–decoder architecture, the proposed approach explicitly integrates theoretical propagation knowledge to enhance learning efficiency and prediction accuracy. Ablation experiments demonstrate that the inclusion of expert knowledge significantly improves the performance of DL-based radio environment DTs. This work highlights the potential of knowledge–data dual-driven DL as a promising direction for advancing radio propagation environment DTs.

## 1. Introduction

Digital Twin (DT) technology has emerged as a transformative concept that bridges the physical and virtual worlds through real-time data synchronization, high-fidelity modeling, and intelligent decision-making. By constructing a dynamic digital replica of a physical system, DTs enable continuous monitoring, simulation, optimization, and predictive analysis across a wide range of domains, including smart manufacturing, autonomous transportation, and next-generation wireless networks [1]. The fundamental principle of DT lies in establishing a bidirectional mapping between physical entities and their digital counterparts, such that the virtual system can accurately reflect, predict, and optimize the behavior of the physical system. In the context of wireless communications, DTs have been increasingly explored to facilitate intelligent resource management in the radio access network (RAN). A dedicated RAN DT can provide a pre-verification environment for intelligent resource management [2]. With the rapid evolution of communication technologies toward the 6G era, DTs are widely recognized as a key enabler for future intelligent and autonomous networks.

Wireless communication networks provide the essential infrastructure for realizing DTs at scale by supporting massive connectivity, low-latency data exchange, and ubiquitous sensing. Conversely, DTs offer powerful tools for network intelligence, enabling real-time emulation of network behavior, adaptive resource allocation, and environment-aware optimization. This mutual reinforcement has attracted considerable research interest in communication-network-oriented DTs, where the accurate digital modeling of the wireless channel becomes a cornerstone for performance evaluation and intelligent control [3]. The propagation of electromagnetic waves determines key network performance indicators such as signal coverage, interference, and throughput. Within this context, the radio propagation environment serves as one of the most fundamental components of communication-oriented DTs. Accurately replicating the radio propagation environment in digital form—referred to as a radio propagation environment DT—is therefore essential for enabling predictive, adaptive, and intelligent wireless communication systems. Particularly in the context of the Internet of Things (IoT) and smart indoor environments, wireless sensor networks (WSNs) form the critical sensory infrastructure. The reliable transmission of sensor data and the effectiveness of multi-sensor fusion depend heavily on robust wireless signal processing within complex propagation environments. Consequently, constructing a high-fidelity radio propagation environment DT is not only a fundamental communication problem but also a crucial prerequisite for the optimal deployment and reliable operation of sensor networks. However, constructing such a DT remains challenging due to the complex interactions between radio signals and environmental elements, including reflection, diffraction, scattering, and shadowing. These interactions result in highly nonlinear and location-dependent propagation characteristics.

To characterize these variations quantitatively, path loss (PL) is a key metric that describes the attenuation of signal power as it propagates through space. Path loss modeling serves as the foundation for analyzing communication coverage, interference estimation, and link budgeting, and thus plays a central role in DT construction for wireless systems [4]. Traditional PL models can be broadly divided into two categories: deterministic models, such as ray tracing (RT), which simulate wave propagation based on geometric optics principles, and empirical statistical models, which approximate propagation behavior using regression or curve-fitting based on measurement data. While deterministic models provide high accuracy, their computational complexity and sensitivity to environmental changes severely limit scalability, making them inadequate for dynamic DTs that require real-time adaptation. Empirical statistical models, on the other hand, offer lower computational cost but often lack generalization capability and spatial fidelity. Moreover, due to the intrinsic nature of electromagnetic wave propagation, PL is a highly complex function of spatial coordinates and environmental configurations [5], further complicating accurate modeling with traditional empirical models.

To overcome these limitations, deep learning (DL) has emerged as a powerful alternative for radio propagation modeling. DL-based approaches leverage a large dataset to automatically learn the mapping from environmental representations (e.g., geometry, material, or imagery) to propagation metrics, including PL. By exploiting high-dimensional feature representations, DL models are capable of capturing complex nonlinear relationships that are difficult to express analytically. Recently, the broader vision of integrating Artificial Intelligence (AI) with DTs for Beyond 5G and 6G networks has been extensively discussed in various industry white papers [6]. For instance, numerous ongoing efforts aim to predict radio propagation directly from spatial map data using deep Convolutional Neural Networks (CNNs), (e.g., the residual network utilized in [7]). While these conventional works highlight the overarching potential of AI-driven radio propagation modeling, most existing algorithmic implementations treat DL as a purely data-driven ‘black box.’ They typically feed raw geographical matrices (such as relative height or distance maps) into the network, relying on the model to implicitly learn complex physical phenomena like shadowing variations from the raw data. Such conventional data-driven models struggle to generalize across diverse physical environments without massive training datasets, highlighting a lack of explicit physical awareness and interpretability.

A promising research direction to address this limitation is the integration of domain or expert knowledge into data-driven learning. This idea has been successfully explored in several fields, such as physics-informed neural networks for fluid dynamics [8] and hybrid physical–data-driven models for remote sensing [9]. However, the systematic incorporation of expert knowledge into DL-based radio propagation environment DT construction remains underdeveloped. In wireless propagation modeling, such expert knowledge may include principles of reflection, diffraction, and attenuation; geometric relationships between transmitters and obstacles; and material-dependent propagation effects. Embedding such knowledge into DL models can guide learning toward physically consistent solutions, thereby improving prediction accuracy, generalization, and interpretability. This motivates a shift from purely data-driven learning to a knowledge–data dual-driven paradigm, which is particularly appealing for constructing reliable radio propagation environment DTs.

Motivated by this gap, this paper focuses on constructing a high-precision radio propagation environment DT through an expert knowledge-infused DL design. Recently, some works [10,11] have begun to explicitly incorporate the occlusion of the transceiver line-of-sight (LoS) path as an input to the DL model, which is a natural extension of differentiating between LoS and non-LoS (NLoS) links. These approaches can be viewed as early attempts at expert knowledge infusion, yet they remain limited in their ability to capture reflection propagation behavior in complex indoor environments.

In this paper, we propose a general methodology that systematically integrates radio propagation theory into the DL pipeline for PL prediction, aiming to support the construction of a high-fidelity radio propagation environment DT. The proposed expert knowledge-infused learning is built upon the widely adopted encoder–decoder architecture, where physical knowledge is encoded in the form of expert knowledge-infused matrices and incorporated as input representations for the DL model. These knowledge-infused matrices—derived from theoretical propagation insights—represent mechanisms such as dominant paths, geometry-dependent attenuation, and material-based signal interactions, thereby complementing raw environmental data. By embedding these expert knowledge-infused matrices into the learning process, the model can efficiently capture implicit propagation laws that are otherwise difficult to learn solely from raw data.

Specifically, the main contributions of this work are summarized as follows:

- We propose an expert knowledge-infused learning method for constructing radio propagation environment DTs, which systematically integrates radio propagation theory into DL–based PL prediction.
- We transform expert knowledge into structured and learnable input representations by designing expert knowledge-infused matrices that encode physically meaningful propagation mechanisms, including penetration and reflection.
- We incorporate the proposed expert knowledge-infused matrices into classic encoder–decoder-based DL architecture as input channels, establishing a knowledge–data dual-driven learning paradigm that enhances generalization capability.
- Through extensive experiments, we demonstrate that expert knowledge-infused matrices play a critical role in maintaining physical consistency and prediction accuracy, especially under incomplete or localized environmental information.

## 2. Problem Formulation

In a RAN-oriented DT, the radio propagation environment serves as a fundamental component that models the radio propagation for performance prediction and intelligent decision-making within the DT. In this section, we formulate the PL prediction problem as the core functionality of the radio propagation environment DT. While a comprehensive radio propagation DT could also encompass parameters like delay and angular spread, this paper strictly focuses on PL prediction as the fundamental first step to validate the proposed knowledge–data dual-driven learning paradigm.

Consider a Tx with the given transmit power $P_{t}$ located at *t* in an indoor environment E. The received power at a receiver (Rx) at location r∈E is given by

(1) $$P_{r}\left(r\right)=P_{t}-P\left(r\right)$$

where P(r) denotes the PL between the Tx at *t* and Rx at *r*, expressed in decibels (dB). From a DT perspective, accurate prediction of P(r) is critical, as it directly determines the digital replica’s ability to reflect the operational state of the wireless network.

It is evident that P(r) is simultaneously dependent on the Tx location *t* and the indoor environment E. To accurately represent the PL between any Tx-Rx pair within the target indoor environment in DT, it is necessary to obtain the map

(2) $$F:\left(E,t\right)\mapsto {\left\{P\left(r\right)\right\}}_{r\in E}.$$

Let F denote the DL-based propagation function that characterizes the spatial distribution of total PL within the target environment. The target PL can be obtained with F, formulated as

(3) P(r)=F(E,t,r),∀r∈E

which corresponds to the classic task of PL prediction and constitutes the core propagation engine of the radio propagation environment DT.

The radio propagation modeling problem was first reformulated as an image regression problem in [12], which addressed it using CNN with encoder–decoder architecture. In [13], the indoor propagation modeling problem was first reformulated as an image regression problem. The objective of this image regression problem is to output the distribution of PL of the indoor environment at the target height. At present, in the field of DL research for indoor radio propagation prediction, CNN with encoder–decoder architecture is widely adopted, and the most common task is to predict the PL distribution on a given height plane. Moreover, the prediction of PL at any point in the 3D space can be transformed into the prediction of PL on the corresponding height plane [14]. Therefore, our DL task focuses on predicting the PL for receivers at a specific target height.

Despite their expressive power, purely data-driven DL models often fail to achieve satisfactory accuracy in practice due to limited model capacity, insufficient training data representativeness, or the lack of explicit physical awareness. We endeavor to improve the learning process by infusing expert knowledge of radio propagation into the DL framework. Specifically, we introduce a function K that extracts expert knowledge-based features from the environment, leading to the following formulation:

(4) $$P\left(r\right)=F\left(K\left(E,t,r\right),E,t,r\right),\forall r\in E$$

In large-scale indoor DTs, directly representing the entire environment E as a single high-resolution image poses challenges in terms of spatial resolution and input tensor size. A practical strategy is to partition the indoor environment E into multiple rectangular regions [15], such that

(5) $$E=E_{1}\cup E_{2}\cup \ldots \cup E_{n}$$

where $E_{i}$ denotes the *i*-th subregion. This partitioning enables the DL model to predict PL distributions within each region independently. Under this setting, the PL prediction task in Equation (3) is reformulated as

(6) $$P\left(r\right)=F\left(E_{i},t,r\right),\forall r\in \left\{E_{i}\right\}$$

which we refer to as the extension task of PL prediction.

**Figure 1:**
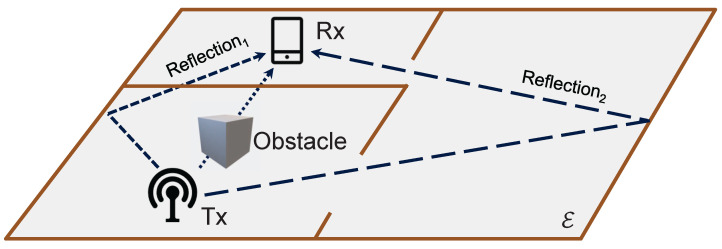
Schematic diagram illustrating significant radio propagation mechanisms (e.g., LoS, reflection, and penetration) in an indoor environment for DT construction.

However, indoor radio propagation is inherently influenced by global environmental structures. Even when both the Tx and Rx are located within the same subregion $E_{i}$, reflection within another region $E_{j}$ may contribute significantly to the received signal power, e.g., Reflection_2_ shown in Figure 1. Accordingly, the expert knowledge–driven analysis must account for the entire indoor environment E. Incorporating this consideration, Equation (4) is transformed as

(7) $$P\left(r\right)=F\left(K\left(E,t,r\right),E_{i},t,r\right),\forall r\in E_{i}$$

In the remainder of this paper, we will introduce the data-driven DL solutions for both the classic PL prediction task in (3) and the extension task in (6), and detail the design of expert knowledge-infused learning corresponding to Equations (4) and (7).

## 3. Data-Driven Deep Learning

In this section, we first introduce the traditional data-driven solutions for the classic task (Equation (3)) as the baseline, detailing the design of the DL models and inputs. Subsequently, we extend these solutions to the subregion-based extension task (Equation (6)).

### 3.1. DL Model

Due to considerations such as computational efficiency, using image-based inputs rather than structured engineering feature tables has become the dominant approach in recent studies. Consequently, the PL prediction task in Equation (3) is naturally formulated as an image-to-image regression problem. CNN employing encoder–decoder architectures, predominantly represented by U-Net [16], has attracted widespread attention for indoor radio propagation prediction.

**Figure 2:**
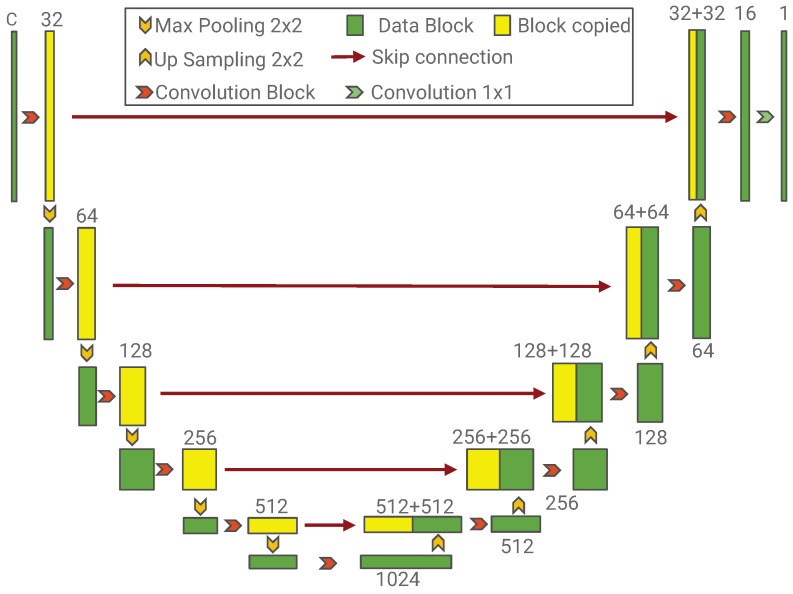
Architecture of the U-Net for PL prediction.

As shown in Figure 2, the U-Net is composed of an encoder and a decoder, which are responsible for feature extraction and reconstruction, respectively. Both the encoder and decoder consist of five layers built from multiple convolution blocks. Each block contains two 3×3 convolution operators, each followed by a Batch Normalization (BN) layer and an activation function ReLU. BN is widely used for regularization and accelerating convergence by normalizing the inputs of each layer to have zero mean and unit variance.

Downsampling is performed using 2×2 max-pooling operation with a stride of 2, effectively reducing the spatial resolution by half. Conversely, upsampling is achieved using 2×2 transposed convolution (ConvTranspose) operation, which can double the spatial dimensions to reconstruct the data layout.

The inputs to the U-Net are matrices with *C* channels. After the initial convolution blocks in the first encoder layer, a feature map with 32 channels is generated. Before reducing the spatial resolution via downsampling, this feature map is preserved and passed to the corresponding decoder layer via a skip connection. As the network depth increases, the spatial resolution of the tensors decreases while the number of channels increases, gradually transforming low-level appearance features into higher-level abstract semantic representations.

Each decoder layer begins with a ConvTranspose operation, followed by convolution blocks to reconstruct the feature representation. The feature map passed from the corresponding encoder layer via the skip connection is concatenated with the upsampled output of the ConvTranspose along the channel dimension. The resulting combined feature map is then fed into the subsequent convolution block. In the final output layer of the decoder, the reconstructed data is integrated to generate the PL distribution map with a single channel.

To verify that proposed expert-learning-infused learning paradigm is not only applicable to a certain DL model, we will also test it on more DL models, including:

- KAUNet: The KAN [17] is more interpretable than MLP, in part because it uses a learnable nonlinear activation function instead of linear changes, which can also be used to replace linear changes in the convolution kernel in U-Net for greater learning ability [18], resulting in KAUNet.
- SDUNet: A widely adopted approach to enlarging the receptive field of CNN is dilated convolutions. Based on this principle, SDUNet incorporate stacked dilated convolutions within a U-Net architecture and has demonstrated effectiveness in radio propagation prediction [13,15,19].

In essence, KAUNet and SDUNet represent two distinct paradigms for enhancing U-Net: KAUNet adopts more learnable convolution, while SDUNet adopts convolutions with larger receptive field.

### 3.2. Model Input

It is widely acknowledged that radio propagation channel characteristics are fundamentally determined by the surrounding environment. Although DL models, such as U-Net, can apply inductive bias and extract abstract features, they do not require the same level of environmental detail as physical simulation methods like RT. In indoor scenarios, the pervasive presence of walls and the enclosed spatial structure lead to complex interactions between electromagnetic waves and the environment, particularly at low frequencies, where propagation phenomena such as penetration, reflection, and diffraction coexist.

**Figure 3:**
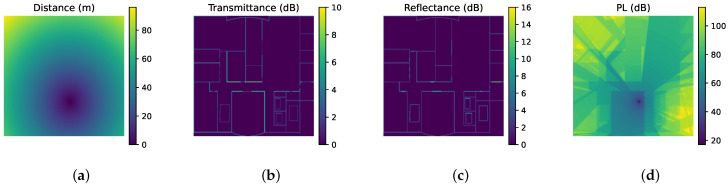
Example of matrices for DL model, visualized as heatmaps: (**a**) D: Distance from each grid cell to the Tx. (**b**) T: Transmittance at normal incidence of each grid cell (0 for air). (**c**) R: Reflectance at normal incidence of each grid cell (0 for air). (**d**) P: GT of PL distribution to be predicted.

We discretize the target indoor environment E into a matrix with a specific step size, where each grid cell represents the minimum spatial resolution for the Tx and Rx positions. To capture the spatial relationships among objects and the antenna array, the distance from each grid cell point to the Tx is calculated and included as an input matrix D, as shown in Figure 3a. For indoor PL prediction, it is crucial to incorporate physical quantities that characterize the electromagnetic properties of objects as input features, commonly the normal-incidence transmittance and expressed in decibels (dB) [15]. Figure 3b,c illustrate the input matrices representing the transmittance and reflectance at each grid, where a value of 0 denotes free space (the absence of obstacles). These matrices are denoted as T and R, respectively.

The three input matrices described above provide comprehensive physical information about the target indoor environment E. The DL model will predict the target output based on input information. The ground truth (GT) of the distribution of PL within E is shown in Figure 3d, denoted as P. Three input matrices and target outputs shown in Figure 3 are the raw data from the publicly available dataset [20], which will be utilized in experimental analysis. Given the DL model, inputs and target outputs, Equation (3) can be formally transformed as

(8) P=F(D,T,R)

### 3.3. Data-Driven DL for Extension Task

For the extension task defined in Equation (6), the indoor environment E is uniformly partitioned into multiple rectangular subregions. In our setting, we directly divide the indoor environment into four non-overlapping rectangular areas evenly, formulated as

(9) $$E=E_{1}\cup E_{2}\cup E_{3}\cup E_{4}$$

**Figure 4:**
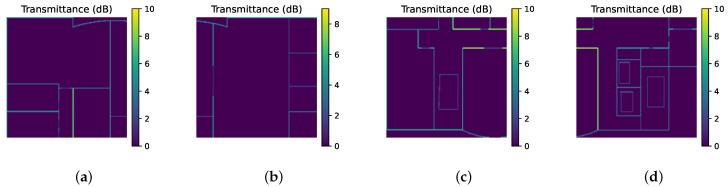
Example of partitioning the matrix T into four subregions, visualized as heatmaps: (**a**) upper-left, (**b**) upper-right, (**c**) lower-left, and (**d**) lower-right.

Accordingly, both the input matrices and output matrices are divided according to the same rules. For example, T shown in Figure 3b is divided into four subregions, i.e., $T_{1},T_{2},T_{3}$, and $T_{4}$, illustrated in Figure 4. The DL model will infer the corresponding result $P_{i}$ based on the inputs from the target area $E_{i}$, formulated as

(10) $$P_{i}=F\left(D_{i},T_{i},R_{i}\right),i\in \left\{1,2,3,4\right\}$$

Theoretically, the regional inferences of the DL model can be executed in parallel. The combined outputs effectively reconstruct the global PL distribution P, rendering this process functionally equivalent to Equation (8) when global information is not strictly required.

## 4. Expert Knowledge-Infused Learning

Although data-driven DL models have demonstrated promising performance in radio propagation prediction, their effectiveness largely depends on the model’s ability to learn complex propagation mechanisms from data implicitly. In practice, whether a DL model can fully capture such mechanisms—especially reflection and penetration effects in indoor environments—remains uncertain and often requires large-scale, highly representative datasets. This limitation can significantly degrade prediction accuracy and generalization capability in practical deployment.

From a learning-theoretic perspective, multi-modal intelligence provides a viable pathway to address this challenge by leveraging complementary sources of information rather than relying solely on raw data [21]. In the context of radio propagation modeling, expert knowledge derived from electromagnetic theory offers structured and physically meaningful information that is difficult for DL models to infer autonomously. Therefore, we argue that explicitly infusing expert knowledge into DL frameworks is essential for constructing high-fidelity radio propagation environment DTs.

In this section, we introduce an expert knowledge-infused learning method, in which expert knowledge is transformed into additional input representations to facilitate the learning process. Specifically, we design two expert knowledge-infused matrices, denoted as $M^{T}$ and $M^{R}$, which encode the physical effects of penetration and reflection, respectively. These matrices serve as structured priors that complement the raw environmental inputs and facilitate knowledge–data dual-driven learning.

In other words, we will complete the expert knowledge construction function K based on the indoor environment E and the transmitter location *t*, formulated as

(11) $$\left\{M^{T},M^{R}\right\}\leftarrow K\left(E,t\right)$$

Accordingly, the DL-based propagation model in (4) is reformulated as

(12) $$P\left(r\right)=F\left(M^{T},M^{R},E,t,r\right),\forall r\in E$$

### 4.1. Expert Knowledge of Penetration

**Figure 5:**
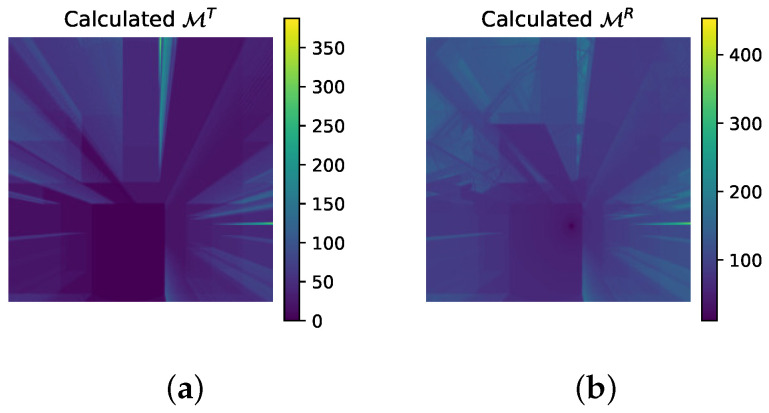
Examples of expert knowledge-infused matrices, visualized as heatmaps: (**a**) $M^{T}$: Matrix encoding cumulative penetration loss along the direct path. (**b**) $M^{R}$: Matrix capturing the dominant reflection path and its associated loss.

In indoor environments, penetration loss caused by obstacles is one of the dominant factors affecting signal attenuation and coverage. Walls, partitions, and other structural elements can significantly attenuate electromagnetic waves, thereby influencing key performance metrics such as cell association and received signal strength. To explicitly model this effect, we construct an expert knowledge-infused matrix $M^{T}$, shown in Figure 5a, which characterizes the cumulative penetration loss along the direct propagation path.

For each candidate Rx location at grid point (x,y), the direct path between the transmitter location *t* and (x,y) is denoted as

(13) $$L_{x,y}=\left\{g_{i}\right\}$$

where each $g_{i}$ represents a discrete grid point $\left(x_{i},y_{i}\right)$ along the line segment connecting *t* and (x,y).

By sampling the transmittance values according to T at $g_{i}$ as $T\left(x_{i},y_{i}\right)$, the cumulative transmittance is calculated as:

(14) $$M^{T}\left(x,y\right)=\sum_{i}T\left(x_{i},y_{i}\right),\;\mathrm{where}\;\left(x_{i},y_{i}\right)\in L_{x,y}.$$

The resulting matrix $M^{T}$ explicitly encodes the penetration effect experienced by the straight line, serving as a physically interpretable feature that reflects obstruction-induced attenuation.

The dataset [20] used in this paper represents indoor environments in a two-dimensional planar form, where the Tx and Rx are located at the same height. Accordingly, we adopt a ray traversal strategy based on the Amanatides-Woo algorithm [22], which efficiently identifies the grid points intersected by a ray in a discrete space through simple linear interpolation. This strategy enables the rapid determination of the path point set $L_{x,y}$ and the construction of the target matrix $M^{T}$. The detailed procedure is summarized in Algorithm 1.

**Algorithm 1** Construction of Expert Knowledge-Infused $M^{T}$
**Require:** Matrix T of size W×H, Tx’s position $t=(x_{t},y_{t})$**Ensure:** Matrix $M^{T}$
  1:Initialize $M^{T}\in R^{W\times H}$ with zeros  2:**for** each grid point (x,y) **do**  3:   Compute direction vector $\left(d_{x},d_{y}\right)\leftarrow \left(x_{t}-x,\;y_{t}-y\right)$  4:   $N\leftarrow \max(|d_{x}|,|d_{y}|)$  5:   **for** n=1 to *N* **do**  6:       $i_{x}\leftarrow x+\left\lfloor n\cdot d_{x}/N\right\rfloor$  7:       $i_{y}\leftarrow y+\left\lfloor n\cdot d_{y}/N\right\rfloor$  8:       **if** $\left(i_{x},i_{y}\right)$ is inside the grid **and** $T(i_{x},i_{y})>0$ **then**  9:            $M^{T}\left(x,y\right)\leftarrow M^{T}\left(x,y\right)+T\left(i_{x},i_{y}\right)$10:       **end if**11:     **end for**12:
**end for**
13:**return** $M^{T}$


To better illustrate this process, consider a specific example where the Tx is located at t=(10,10). Given a target grid point (x,y)=(10,50), we cast a ray from (10,10) to (10,50). Suppose this ray intersects two obstacles located at grid points (10,20) and (10,30), with T(10,20)=3dB and T(10,30)=5dB, respectively, while all other grid points along the path are free space. As the Algorithm 1 traverses the discrete points along the line segment, it accumulates these transmittance values as $M^{T}\left(10,50\right)=T\left(10,20\right)+T\left(10,30\right)=8\;\mathrm{dB}$. This value explicitly informs the DL model that the signal could be attenuated by these specific obstacles. This approach offers a computationally efficient approximation of penetration effects while maintaining sufficient physical fidelity for learning purposes.

It is worth noting that the design of the expert knowledge-infused matrix is not limited to the specific dataset used in this paper. Although the Algorithm 1 is based on 2D floor plans, the methodology can be readily extended to three-dimensional environments by incorporating height and volumetric information.

Regarding the computational cost, the generation of both matrices is highly efficient. As detailed in Algorithm 1, constructing $M^{T}$ requires traversing the grid cells along the direct path, yielding a linear time complexity of O(N) per target Rx, where $N=\max(|d_{x}|,|d_{y}|)$ represents the grid distance between the Tx and Rx. Subsequently, generating $M^{R}$ only evaluates the candidate reflection points identified during the $M^{T}$ traversal. If *K* obstacles are intersected (K≤N), the search complexity for the dominant reflection is merely O(K). This lightweight, linear scaling guarantees that extracting such expert knowledge representations introduces negligible latency, perfectly aligning with the rapid real-time inference requirements of practical radio propagation DTs.

### 4.2. Expert Knowledge of Reflection

In addition to penetration, reflection plays a critical role in indoor radio propagation, particularly in sub-6 GHz frequency bands. Due to multiple interactions between electromagnetic waves and surrounding obstacles, reflected paths can contribute significantly to the received signal power. Among these, single-reflection paths often dominate, as higher-order reflections typically incur excessive attenuation.

In radio propagation analysis, the path associated with the minimum PL is typically identified as the dominant path [23]. Following this concept, we define the dominant reflection path as the single-reflection path with the minimum PL, which corresponds to the most significant reflected propagation component. Based on this concept, we construct an expert knowledge-infused matrix $M^{R}$ illustrated in Figure 5b, which captures the most influential reflected component at each Rx location.

For an interested grid point (x,y), the matrix $M^{T}$ indicates that it has received the signal along the direct path $L_{x,y}$, with the corresponding loss given by:

(15) $$P_{x,y}^{L}=FSPL_{x,y}^{L}+M^{T}\left(x,y\right),$$

where $M^{T}\left(x,y\right)$ is the cumulative transmittance experienced along the direct path and $FSPL_{x,y}^{L}$ is the free-space path loss given by:

(16) $$FSPL_{x,y}^{L}=20\log_{10}\left(4\pi \left(D\left(x,y\right)+\epsilon_{d}\right)f/c_{0}\right)$$

where D(x,y) is the distance from the interested grid point (x,y) to Tx *t* according to matrix D, $\epsilon_{d}$ is a small positive number to avoid singularity, *f* is the frequency, and $c_{0}$ is the speed of light.

Assuming an arbitrary grid point receives a signal through a dominant reflection path originating from the reflecting surface at $\left(x_{r},y_{r}\right)$, the associated loss along this specific path is formulated as follows:

(17) $$P_{x,y}^{R}=Ref_{\left(x_{r},y_{r}\right)}+FSPL_{x,y}^{R}+T_{x,y}^{R},$$

where $Ref_{x_{r},y_{r}}$ denotes the reflection loss incurred during the reflection process. $FSPL_{x,y}^{R}$ represents the free-space path loss calculated based on the total distance of the reflection path from the Tx *t* to (x,y) via $\left(x_{r},y_{r}\right)$, and $T_{x,y}^{R}$ is the cumulative transmittance along this reflected path.

Among all candidate reflection points, the one yielding the minimum PL is selected to construct $M^{R}\left(x,y\right)$ as

(18) $$M^{R}\left(x,y\right)=-10\log_{10}\left(10^{-P_{x,y}^{L}/10}+10^{-P_{x,y}^{R}/10}\right),$$

where the transmit power $P_{t}$ in Equation (1) is omitted. This omission is intentional, as the transmission power is typically determined by the resource scheduling strategy based on the constructed radio propagation environment DT, and is therefore unknown at the stage of PL prediction. Although radio propagation calculations without explicit transmission power may introduce approximation errors, it should be emphasized that the purpose of constructing $M^{R}$ is not to perform a complete ray-tracing-based simulation. Instead, $M^{R}$ is designed to infuse expert knowledge of radio reflection into the DL model, thereby facilitating its ability to learn reflection-related propagation characteristics. We expect that, during training, the DL model can effectively combine these knowledge-driven priors with data-driven learning to improve prediction accuracy.

Traditional ray-tracing engines frequently rely on the shooting and bouncing ray (SBR) technique [24]. In such deterministic simulators, the computational overhead exhibits factorial growth relative to the predefined maximum interaction limits. Specifically, the complexity scales proportionally to $\left(N_{R}+N_{T}+1\right)!/\left(N_{R}!N_{T}!\right)$, with $N_{R}$ and $N_{T}$ denoting the upper bounds for reflections and transmissions, respectively. By contrast, our $M^{R}$ formulation restricts the analysis to a single reflection event (i.e., $N_{R}=1$), thereby ensuring a highly acceptable computational footprint for practical DT deployments. Furthermore, diffraction is also ignored in the input matrices for similar complexity reasons, leaving it to the DL model to infer from the training data.

The construction of $M^{R}$ inherently relies on D,R,T, and $M^{T}$ as prerequisites. Therefore, $M^{R}$ is acceptable when working on the dataset [20] that lack explicit 3D geometry. During the computation of $M^{T}$ via Algorithm 1, potential reflection points $\left(x_{r},y_{r}\right)$ are already identified, alongside their distances to the Tx *t* and the sum of $T(x_{i},y_{i})$ from $\left(x_{r},y_{r}\right)$ to *t*. Consequently, computing the remaining reflection path attributes (e.g., the distance and cumulative transmittance from $\left(x_{r},y_{r}\right)$ to the Rx *r*) simply requires geometric derivation based on the incident angle at $\left(x_{r},y_{r}\right)$. Now, both $FSPL_{x,y}^{R}$ and $T_{x,y}^{R}$ in Equation (17) are obtained. We pick up the reflection with minimum $P_{x,y}^{R}$ to generate $M^{R}\left(x,y\right)$. The overall procedure for constructing the expert knowledge-infused matrix $M^{R}$ is summarized in Algorithm 2.

**Algorithm 2** Construction of Expert Knowledge-Infused $M^{R}$
**Require:** Environment matrices T, R, distance matrix D, Tx position $t=(x_{t},y_{t})$**Ensure:** Matrix $M^{R}$
  1:Initialize $M^{R}\in R^{W\times H}$ with zeros  2:**for** each grid point (x,y) **do**  3:     Compute direct-path loss $P_{x,y}^{L}$ using D and T  4:     Initialize reflected-path loss $P_{x,y}^{R}\leftarrow +\infty$  5:     **for** each candidate reflection point $\left(x_{r},y_{r}\right)$ **do**  6:         Estimate reflection loss at $\left(x_{r},y_{r}\right)$ using R  7:         Compute reflected-path distance via $\left(x_{r},y_{r}\right)$  8:         Accumulate transmittance along the reflected path using T  9:         Evaluate reflected-path loss $P_{x,y}^{R}$10:     **end for**11:     Select the minimum $P_{x,y}^{R}$ as the loss of dominant reflection path12:     Combine $P_{x,y}^{L}$ and $P_{x,y}^{R}$ to obtain $M^{R}\left(x,y\right)$13:
**end for**
14:**return** $M^{R}$


### 4.3. Expert Knowledge-Infused Learning for Extension Task

We hypothesize that expert knowledge-infused learning can achieve superior performance compared to traditional data-driven DL for the classic task. With the expert knowledge-infused matrices introduced above, the data-driven solution Equation (8) is transformed into a knowledge–data dual-driven solution, formulated as

(19) $$P=F(M^{T},M^{R},D,T,R)$$

In the condition that the model takes $D_{i},T_{i},R_{i}$ as the inputs, the expert knowledge-based analysis could work on D,T,R and result in the expert knowledge-infused matrices of target area $E_{i}$, i.e., $M_{i}^{T}$ and $M_{i}^{R}$. Similar to the adjustment in Section 3.3, the expert knowledge-infused learning for the extension task is formulated as

(20) $$P_{i}=F\left(M_{i}^{T},M_{i}^{R},D_{i},T_{i},R_{i}\right),i\in \left\{1,2,3,4\right\}$$

## 5. Numerical Results

In this section, we will complete the implementation of expert knowledge-infused learning on different DL models for both classic task and extension task. The corresponding prediction results are then compared with the traditional data-driven solution to evaluate the effectiveness of the knowledge–data dual-driven solution.

### 5.1. Experiment Settings

Experiments are conducted on the *Indoor Radio Map Dataset* [20], which provides high-fidelity indoor propagation data generated via ray-tracing simulations. A total of 1050 samples are selected, which closely resemble realistic indoor scenarios, ensuring the practical relevance of the evaluation. The carrier frequency is 868 MHz, as lower-frequency signals generally experience slower attenuation, leading to more pronounced multipath effects. Both Txs and Rxs are positioned at a height of 1.5 m. The spatial grid resolution across all radio maps is uniformly established at 0.25 m, matching the discretization granularity required for accurate DT modeling. Omnidirectional antennas are assumed at both the Tx and Rx. To closely emulate complex real-world indoor wave propagation, the ray-tracing simulation was configured to permit a maximum of 8 reflections, 10 transmissions, and 2 diffraction events per ray. Regarding data partitioning, the available samples were randomly divided into training and testing subsets utilizing a standard 9:1 ratio.

To evaluate the generality of the proposed expert knowledge-infused learning framework, three representative encoder–decoder-based DL models are considered: U-Net, SDUNet, and KAUNet. All input and output matrices are normalized to the range [0,1] to stabilize training. Supervised training is performed using the Adam optimizer [25] with an initial learning rate of $10^{-3}$, together with a cosine annealing learning rate scheduler [26]. The batch size is set to B=2, and all models are trained for 200 epochs.

Experiments are carried out on the system equipped with an NVIDIA RTX 4090 GPU and an Intel Xeon Gold 6430 CPU. The root mean square error (RMSE) is adopted as the training loss, given by:

(21) $$RMSE=\sqrt{{\sum_{b=1}^{B}\sum_{i=1}^{H}\sum_{j=1}^{W}\left({y_{b}}^{DL}\left(i,j\right)-{y_{b}}^{GT}\left(i,j\right)\right)}^{2}\;/\;BHW}$$

where ${y_{b}}^{DL}\left(i,j\right)$ is the predicted value at the *i*-th row and *j*-th column of the *b*-th sample, ${y_{b}}^{GT}\left(i,j\right)$ is the GT at the *i*-th row and *j*-th column of the *b*-th sample. Since the output is a 2D PL distribution with a single channel, we omit the channel dimension for simplicity. The mean absolute error (MAE) is adopted as the evaluation metric. These settings ensure that the evaluation focuses on the impact of expert knowledge-infused representations, rather than differences in training protocols or optimization strategies.

### 5.2. Results of Classic Task

This subsection evaluates the effectiveness of the proposed expert knowledge-infused learning framework on the classic indoor PL prediction task. By systematically varying the combinations of input matrices, we aim to examine whether expert knowledge-infused matrices can consistently improve learning accuracy across different DL models. All matrices are scaled to a uniform size H×W=512×512 without the loss of resolution.

To establish a baseline for performance evaluation, we first implement the empirical model recommended in Equation (1) of ITU-R P.1238-11 [27]. By fitting the empirical model to the training dataset, we obtain α≈4.01, β≈23.54, and γ≈15.32. However, this empirical baseline yields a relatively high MAE of approximately 7.5 dB on both the training and test sets. This performance indicates that empirical models lack the granularity required to capture complex indoor radio propagation phenomena, which is critical for high-fidelity DTs.

As shown in Table 1, we utilize various combinations of input matrices to train different DL models, and analyze the role of expert knowledge-infused matrices by comparing their prediction MAEs. Since D provides essential spatial correlation information regarding wireless signal propagation, it is retained in every combination.

Traditional data-driven learning using the conventional inputs D,T,R yields the highest MAE across all three models, indicating that learning complex indoor propagation mechanisms solely from raw environmental data remains challenging.

Introducing the expert knowledge-infused matrix $M^{T}$ leads to a substantial reduction in MAE for all models, regardless of whether $M^{T}$ replaces T or is used jointly with it. This aligns with the general consensus in multi-modal learning that superior performance is achieved when $M^{T}$ is used jointly with T.

Although $M^{T}$ is derived from T and D, and inherently contains less information than the full transmittance matrix, it explicitly encodes cumulative penetration effects along dominant propagation directions. This structured representation significantly facilitates model learning, particularly under limited data conditions.

$M^{T}$ derived from D and T only provides the transmittance along the line $L_{x,y}$. In other words, the information provided by $M^{T}$ is less than that of T. From a theoretical perspective, a sufficiently expressive DL model might be expected to implicitly learn penetration effects from T and D, rendering $M^{T}$ redundant. However, such an idealized model is rarely achievable in practice. The effectiveness of $M^{T}$ can be attributed to its explicit encoding of cumulative penetration effects, which are otherwise difficult for DL models to infer implicitly from sparse transmittance information. This also explains why all DL models achieve better performance when $M^{T}$ replaces T.

Further improvements are observed when the reflection-knowledge-infused matrix $M^{R}$ is introduced. The inclusion of $M^{R}$ consistently enhances prediction accuracy across all models, demonstrating its effectiveness in guiding the learning of reflection-related propagation characteristics. It is worth emphasizing that $M^{R}$ does not aim to provide an exact calculation of reflected PL. Instead, it serves as a physically informed indicator of dominant reflection relevance, guiding the DL model toward spatial regions with potentially significant reflected contributions.

**Figure 6:**
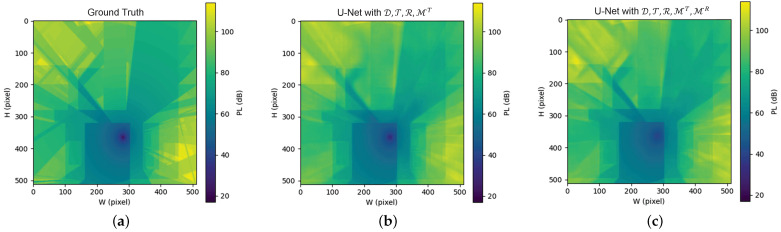
Results of the classic task: (**a**) GT of the PL distribution. (**b**) Predicted PL by U-Net using inputs D, T, R and $M^{T}$. (**c**) Predicted PL by U-Net using inputs D, T, R, $M^{T}$ and $M^{R}$.

As shown in Figure 6c, the upper-left region of the U-Net output captures substantially richer reflection phenomena compared with Figure 6b. Notably, Figure 6c visually indicates that the U-Net has learned additional second-order reflection information, even though such information is not explicitly provided through the input matrix $M^{R}$. This observation suggests that dominant reflection path information infused via $M^{R}$ can effectively guide the learning process, enabling the model to infer more complex reflection behaviors beyond the infused expert knowledge.

We then examine the performance of each model under different input combinations, with D, $M^{T}$, and $M^{R}$ fixed. When R is incorporated as an additional input, the MAE of all three models on the test set is reduced, achieving performance comparable to that obtained using five matrix inputs. This observation suggests that $M^{R}$ mainly captures the dominant reflection path information. Its key role is to assist the DL model in identifying which Rx locations are influenced by reflected signals from a reflection point, rather than directly providing the exact PL. Consequently, when R is included, the DL models can learn the reflection loss implicitly during training and are even capable of inferring the impact of multiple reflection paths.

When T is included as an additional input without R, the performance of all three DL models deteriorates. We attribute this performance drop to two factors. First, T provides limited additional value for learning radio propagation when $M^{T}$ is already present. Second, unlike the other dense matrices, T is highly sparse, which increases the learning burden on the convolution operators. Consequently, the performance on the test set deteriorates. Furthermore, the sparsity of T exhibits a more pronounced negative impact on dilated convolutions, which explains the increased training MAE of SDUNet compared to the combination of D, $M^{T}$ and $M^{R}$.

Overall, the results demonstrate that incorporating expert knowledge-infused matrices consistently reduces the MAE for all evaluated models. This confirms that infusing radio propagation knowledge into the learning process is beneficial.

### 5.3. Results of Extension Task

We further evaluate the proposed expert knowledge-infused learning method on the extension task, where the indoor environment is divided into multiple subregions. This setting is particularly challenging, as radio propagation at a given location may be significantly influenced by obstacles and reflections outside the local region. Here, the partitioning of the dataset into training and testing sets remains unchanged; the assignment of each sample corresponding to any indoor environment E is identical to the classic task. Since the indoor environment E should be divided into four $E_{i}$ according to Section 3.3, the size of all input matrices (e.g., $D_{i}$, $M_{i}^{T}$) is scaled to H×W=256×256. As before, $D_{i}$ is retained in every combination.

**Figure 7:**
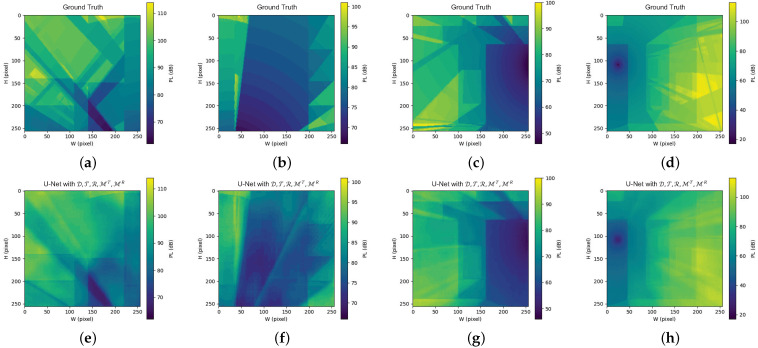
Comparison of PL distributions for the partitioned indoor environment in the extension task. (**a**–**d**) GT for the four partitioned regions. (**e**–**h**) Corresponding U-Net prediction results for the same regions.

As shown in Figure 7, the Tx is located within the subregion corresponding to Figure 7d, while reflection is captured within Figure 7e. It is worth noting that an unexpected line traversing from the lower-left corner to the upper-right corner appears in Figure 7f. Further analysis reveals that this is caused by the coarse spatial resolution of the original environmental information in the dataset, which leads to unintended penetration effects during the construction of the $M^{T}$. In practical DT deployments, environmental mapping is typically acquired via environmental sensors (e.g., LiDAR, depth cameras, or spatial sensing arrays). The spatial resolution and measurement accuracy of these sensing technologies directly dictate the quality of the input matrices, which in turn significantly influences the AI model’s prediction fidelity. Such unexpected errors explicitly highlight the dependency of AI-driven framework on high-precision sensing technology, and can be effectively mitigated by employing more accurate 3D sensor data to represent indoor scenes.

As shown in Table 2, traditional data-driven learning suffers from severe performance degradation in the extension task, with MAE exceeding 7 dB. This result highlights the limitation of relying solely on local environmental information when global propagation effects are present. To achieve sufficient accuracy for a radio propagation DT using purely data-driven models, the training set size would need to be drastically increased (e.g., over 500,000 samples as in [15]) to properly fit the complex propagation effects from raw data.

In contrast, incorporating the expert knowledge-infused $M_{i}^{T}$ and $M_{i}^{R}$ leads to substantial performance improvements. By encoding global propagation knowledge into locally computable representations, the proposed expert knowledge-infused matrices effectively compensate for the loss of non-local information introduced by spatial partitioning.

When the expert knowledge-infused matrices are adopted, either as replacements or supplements, all three models exhibit significant performance improvements. Notably, compared to using all five matrices as inputs, the accuracy of the SDUNet decreased significantly when omitting $T_{i}$. We attribute this to SDUNet’s reliance on dilated convolutions; while they expand the receptive field and aggregate more spatial information, they do not inherently enhance the kernel’s ability to analyze highly complex, infused inputs. As a result, both U-Net and KAUNet achieve more substantial improvements and lower MAEs than SDUNet under the expert knowledge-infused learning paradigm.

On the other hand, KAUNet’s convolution kernels with greater learning ability focus on the 3×3 regions to extract radio propagation knowledge. This indicates that stronger local modeling capability is particularly well-suited for expert knowledge-infused learning.

These results provide insights into model design under the expert knowledge-infused learning paradigm, suggesting that improving local modeling expressiveness is more effective than simply expanding the receptive field when expert knowledge is incorporated. Owing to the strong spatial correlation among adjacent locations in radio propagation, local dependencies can be recursively extended to long-range interactions, especially when guided by expert knowledge. Therefore, architectures emphasizing local modeling are more suitable for exploiting expert knowledge than those relying primarily on enlarged receptive fields.

## 6. Conclusions

This paper investigated the construction of radio propagation environment DTs by infusing expert knowledge into DL-based PL prediction. An expert knowledge-infused learning method was proposed, in which physically meaningful propagation knowledge is encoded in the form of matrices and infused into the encoder–decoder-based DL architectures. By modeling penetration and reflection effects, the proposed approach enhances the physical awareness of data-driven propagation learning. Experimental results demonstrate that the proposed method consistently improves PL prediction accuracy across different network architectures. Moreover, the results indicate that expert knowledge plays a crucial role in preserving global physical consistency under localized or incomplete environmental information. It fundamentally enhances the model’s generalization capability across varying layouts even with limited training data, which is particularly important for practical DT deployment.

While this study validates the knowledge–data dual-driven paradigm using high-fidelity 2D ray-tracing data, several avenues remain for future research. First, we plan to extend the framework to full 3D volumetric scenarios and fine-tune the models with large-scale empirical measurements to ensure enhanced reliability and robustness in practical deployments. Second, exploring the integration of our expert knowledge matrices with emerging physics-informed neural networks presents a promising direction. Finally, evaluating the downstream system-level impact of this PL engine—particularly its practical applications in AI-driven sensor network optimization, such as optimal sensor node placement, energy-efficient power control, and signal processing adaptation under complex propagation conditions—will be a primary focus of our subsequent investigations to further bridge the gap between radio propagation modeling and intelligent sensing systems.

## Figures and Tables

**Table 1:** MAE (dB) of DL models under different input combinations for classic task.

Inputs	U-Net		SDUNet		KAUNet	
	Train Set	Test Set	Train Set	Test Set	Train Set	Test Set
D, T, R	4.653	4.844	3.780	3.875	3.231	3.526
D, R, $M^{T}$	2.422	3.056	2.850	3.062	1.691	2.328
D, T, R, $M^{T}$	1.367	2.313	2.500	2.768	1.343	2.045
D, T, R, $M^{T}$, $M^{R}$	1.392	2.268	2.122	2.429	1.027	1.928
D, $M^{T}$, $M^{R}$	1.949	2.726	2.126	2.723	1.284	2.240
D, R, $M^{T}$, $M^{R}$	1.449	2.272	1.908	2.466	1.060	1.947
D, T, $M^{T}$, $M^{R}$	1.758	2.831	2.341	2.859	1.317	2.506

**Table 2:** MAE (dB) of DL models under different input combinations for extension task.

Inputs	U-Net		SDUNet		KAUNet	
	Train Set	Test Set	Train Set	Test Set	Train Set	Test Set
$D_{i}$, $T_{i}$, $R_{i}$	6.363	6.925	7.176	7.293	4.967	5.067
$D_{i}$, $M_{i}^{T}$, $M_{i}^{R}$	4.360	4.413	4.281	4.396	2.535	2.968
$D_{i}$, $R_{i}$, $M_{i}^{T}$, $M_{i}^{R}$	3.122	3.241	3.932	4.002	2.227	2.815
$D_{i}$, $T_{i}$, $R_{i}$, $M_{i}^{T}$, $M_{i}^{R}$	2.390	2.900	3.233	3.340	2.223	2.674

## Data Availability

The original contributions presented in this study are included in the article. Further inquiries can be directed to the corresponding author.

## Abbreviations

The following abbreviations are used in this manuscript:

DT	Digital Twin
DL	Deep Learning
PL	Path Loss
CNN	Convolutional Neural Network
RT	Ray Trace
